# Progressive Purpura in Microscopic Polyangiitis: A Case Report

**DOI:** 10.7759/cureus.93569

**Published:** 2025-09-30

**Authors:** Hiroaki Watanabe, Kosuke Ishizuka, Megumi Hiida, Kenya Ie, Chiaki Okuse

**Affiliations:** 1 Department of General Internal Medicine, Kawasaki Municipal Tama Hospital, Kawasaki, JPN; 2 Department of General Internal Medicine, St. Marianna University School of Medicine, Kawasaki, JPN; 3 Department of General Medicine, Yokohama City University School of Medicine, Yokohama City, JPN; 4 Department of Rheumatology and Allergology, Kawasaki Municipal Tama Hospital, Kawasaki, JPN

**Keywords:** anca-associated vasculitis, eosinophilic granulomatosis with polyangiitis, granulomatosis with polyangiitis, microscopic polyangiitis, purpura

## Abstract

Purpura is one of the most common cutaneous manifestations of microscopic polyangiitis (MPA), typically presenting as palpable lesions on the lower extremities due to leukocytoclastic vasculitis. Although nonspecific, these findings often appear early and may provide important diagnostic clues before life-threatening organ involvement becomes evident.

A 72-year-old woman presented with a two-month history of dry cough and a one-month history of bilateral femoral myalgia. Physical examination revealed fine crackles in the bilateral lower dorsal lung fields and localized purpura on her right lower extremity. Grasping of the limbs elicited tenderness in the bilateral quadriceps femoris, tibialis anterior, and extensor hallucis longus. A plain chest computed tomography scan revealed interstitial opacities in the subpleural regions of both lungs. Laboratory tests showed a hemoglobin level of 9.5 g/dL and a C-reactive protein level of 8.81 mg/dL. Urinalysis revealed proteinuria (0.32 g/gCr) without hematuria. Five days after the initial presentation, palpable purpura was noted on both lower extremities, along with tingling pain radiating from the dorsal feet to the toes. Myeloperoxidase-anti-neutrophil cytoplasmic antibody (MPO-ANCA) was elevated at 16.4 IU/mL. Nerve conduction studies revealed an axonal degeneration pattern in the bilateral lower limbs. A skin biopsy demonstrated leukocytoclastic vasculitis within the dermis, indicating vasculitis. Based on these findings, MPA was diagnosed. Treatment with prednisolone and azathioprine resulted in the rapid resolution of the purpura. This case underscores the importance of recognizing unilateral or progressive purpura as an early diagnostic clue of MPA, even before renal or pulmonary involvement becomes evident.

## Introduction

Microscopic polyangiitis (MPA) is a pauci-immune necrotizing small-vessel vasculitis strongly associated with myeloperoxidase antineutrophil cytoplasmic antibody (MPO-ANCA). MPO-ANCA activates neutrophils, leading to endothelial injury and leukocytoclastic vasculitis [1-2]. The disease concept of ANCA-associated vasculitis (AAV) encompasses three conditions: granulomatosis with polyangiitis (GPA), MPA, and eosinophilic granulomatosis with polyangiitis (EGPA) [3]. AAV presents with a wide variety of clinical manifestations, and its diagnosis can be challenging, as ANCA negativity does not necessarily exclude the disease.

Cutaneous involvement, particularly purpura due to leukocytoclastic vasculitis, is among the most frequent findings in MPA, reported in approximately half of patients [4]. These lesions are typically palpable, symmetrically distributed on the lower extremities, and provide an important early diagnostic clue. The differential diagnosis includes immunoglobulin A (IgA) vasculitis, infection-related vasculitis, drug-induced vasculitis, and connective tissue disease-associated vasculitis [5]. Diagnostic confirmation relies on a combination of ANCA serology, skin biopsy, and systemic evaluation for renal, pulmonary, and neurologic involvement [6]. Management is determined by severity: for organ- or life-threatening disease, induction with glucocorticoids plus rituximab or cyclophosphamide is recommended; for maintenance, rituximab is preferred, with azathioprine as an alternative and mycophenolate considered in selected cases [7]. Complications such as rapidly progressive glomerulonephritis, diffuse alveolar hemorrhage, and interstitial lung disease further underscore the prognostic significance of early recognition of evolving purpura [8-9]. Against this background, we present a 72-year-old woman whose evolving cutaneous purpura served as the pivotal clue to an early diagnosis of MPA before major organ involvement.

## Case presentation

**Figure 1:**
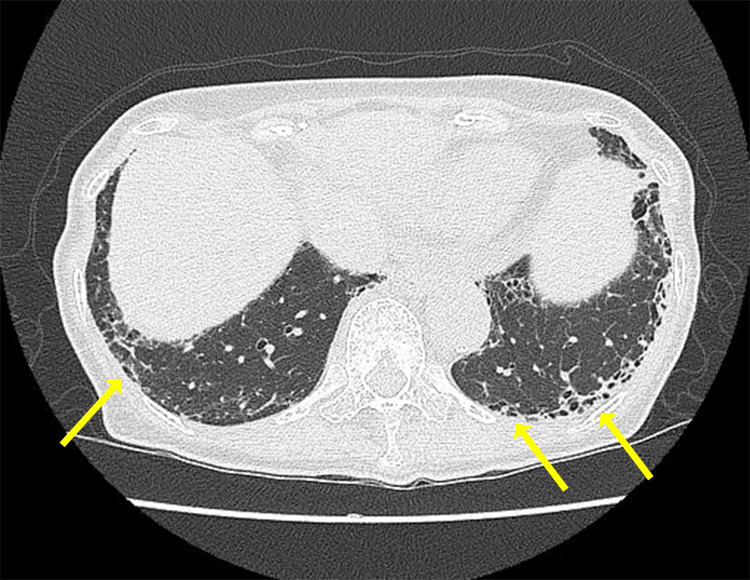
Computed tomography (CT) of the chest Reticular opacities are observed in the peripheral lung bases, consistent with mild interstitial pneumonia.

A 72-year-old woman presented with a two-month history of dry cough and a one-month history of bilateral femoral myalgia. The patient had exertional pain such as “feeling fatigued when climbing stairs” and “having difficulty rising from the floor.” Her medical history was significant for diabetes mellitus. Her medications included sitagliptin, gliclazide, metformin, and rosuvastatin. Vital signs were within normal limits. Physical examination revealed fine crackles in the bilateral lower dorsal lung fields and localized painless nonpalpable purpura on her right lower extremity. The heliotrope rash, Gottron’s sign, and the V-sign were not observed. Grasping of the limbs elicited tenderness in the bilateral quadriceps femoris, tibialis anterior, and extensor hallucis longus. Muscle weakness was observed with manual muscle testing, showing quadriceps 4/4 and hamstrings 4/4. A plain chest computed tomography scan revealed interstitial opacities in the subpleural regions of both lungs (Figure 1), predominantly in the left lung, suggestive of the usual interstitial pneumonia (UIP) pattern.

No distinct neoplastic lesions were identified. Laboratory tests showed a hemoglobin level of 9.5 g/dL and a C-reactive protein level of 8.81 mg/dL (Table 1). No elevation in creatine kinase was observed. Urinalysis revealed proteinuria (0.32 g/gCr) without hematuria.

**Table 1:** Laboratory findings

Variable	At presentation	Reference range (adults)
Erythrocyte count (10^6/μl)	3.23	3.86-4.92
Hemoglobin (g/dl)	9.5	11.6-14.8
Leukocyte count (10^3/mm^3)	11	3.3-8.6
Platelet count (10^3/μl)	311	158-348
Urea nitrogen (mg/dl)	9.8	Aug-20
Creatinine (mg/dl)	0.52	0.46-0.79
Glucose (mg/dl)	161	73-109
Hemoglobin A1c (%)	6.9	4.9−6.0
Albumin (g/dl)	2.7	4.1-5.1
Alanine aminotransferase (U/liter)	12	Jul-23
Aspartate aminotransferase (U/liter)	18	13-30
Alkaline phosphatase (U/liter)	76	38-113
γ-Glutamyltransferase (U/liter)	14	Sep-32
Creatine kinase (U/liter)	41	41-153
Erythrocyte sedimentation rate (mm/hr)	71	< 15
C-reactive protein (mg/dl)	8.81	< 0.14
KL-6 (U/ml)	335	< 500
Antinuclear antibody	40	Negative at 1:40 dilution
Rheumatoid factor (IU/ml)	161	< 15
Proteinase3-antineutrophil cytoplasmic antibody (PR3-ANCA) (IU/ml)	1.6	< 2
Myeloperoxidase anti-neutrophil cytoplasmic antibody (MPO-ANCA) (IU/ml)	16.4	< 3.5
Anti-citrullinated cyclic peptide (U/ml)	1.1	< 4.4
Anti-RNP antibody (U/ml)	8.2	< 9.9
Anti-Smith antibody (U/ml)	<1	< 9.9
Anti-SS-A antibody (U/ml)	<1.0	< 9.9
Anti-SS-B antibody (U/ml)	1.1	< 9.9
Anti-Scl-70 antibody (U/ml)	<1	< 9.9
Anti-aminoacyl-tRNA synthetase antibody (U/ml)	5.2	< 24.9
Anti-Mi-2 antibody (index)	<5	< 53
Anti-TIF1-γ antibody (index)	<5	< 32
Anti-MDA5 antibody (index)	<4	< 32
Urine Analysis		
Sediment		
Red cells	1-4/HPF	-
White cells	1-4/HPF	-
Squamous cells	1-4/HPF	-
Hyaline casts	1+	-
Protein (mg/dl)	29	< 20
Creatinine (mg/dl)	91.3	NA

**Figure 2:**
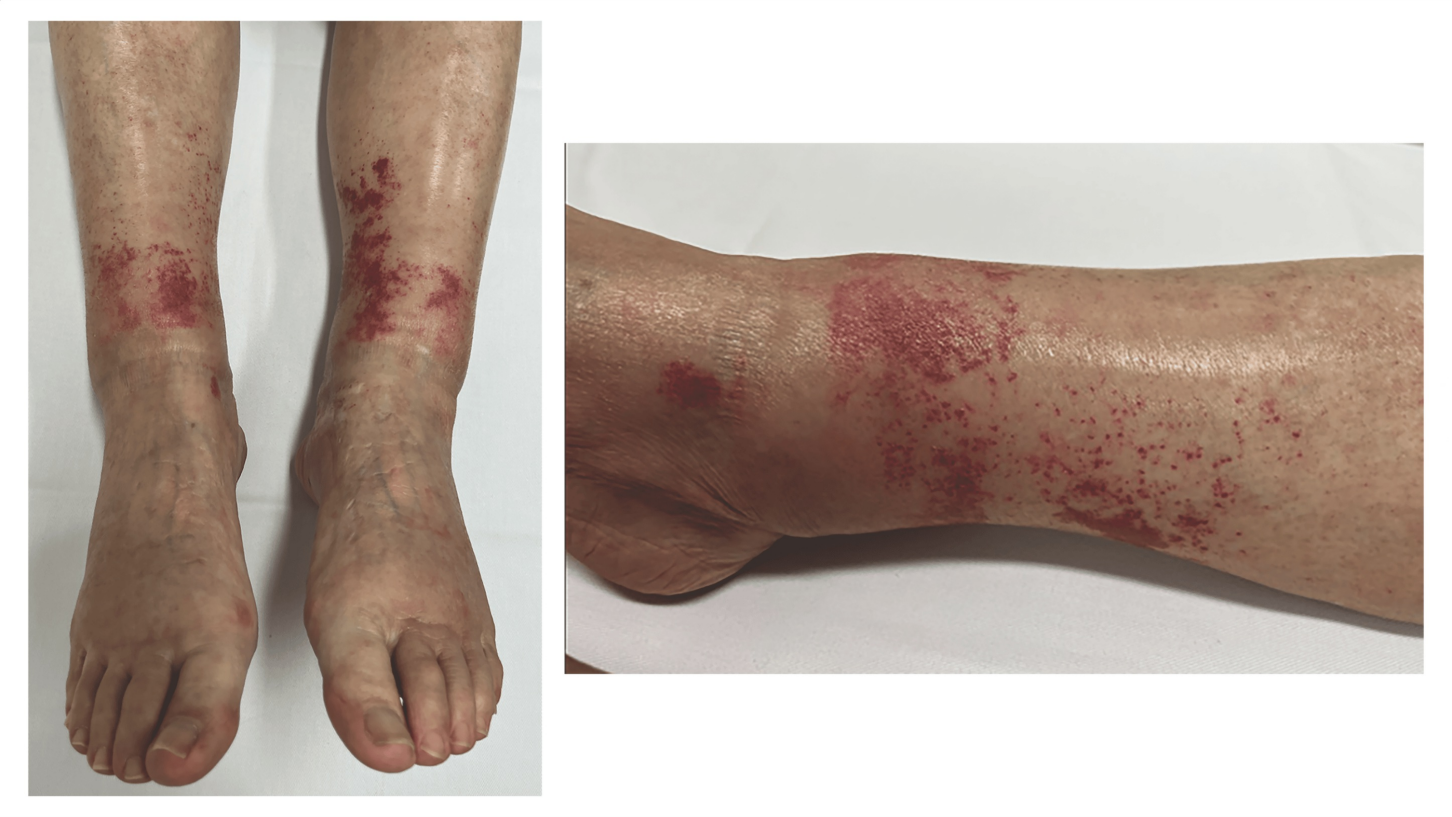
Five days after the initial presentation, palpable purpura was noted on both lower extremities, along with tingling pain radiating from the dorsal feet to the toes.

Five days after the initial presentation, painless palpable purpura was noted on both lower extremities, along with tingling pain radiating from the dorsal feet to the toes (Figure 2).

**Figure 3:**
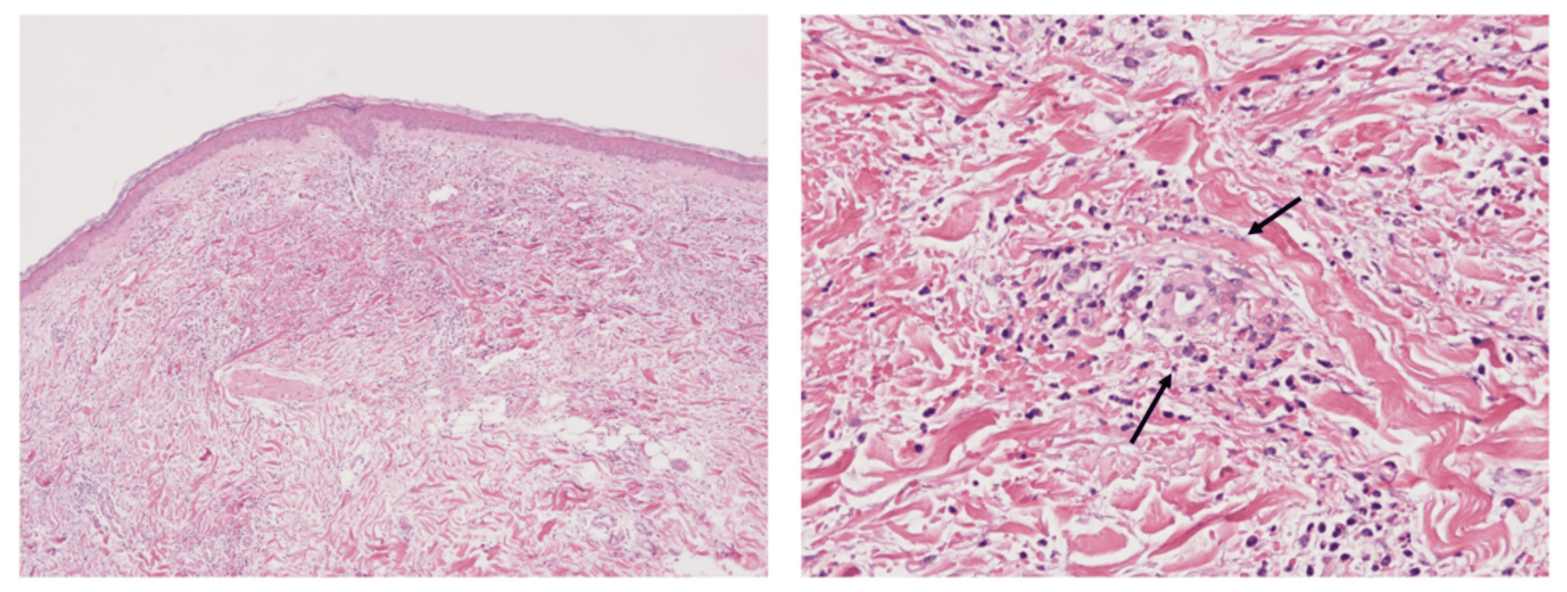
Pathological findings Low-power field (left) and high-power field (right) of a skin biopsy specimen from the left extremity (hematoxylin–eosin staining). Dense inflammatory infiltrates with leukocytoclastic changes (↑) are seen in the dermis surrounding small vessel walls. These changes are characterized by nuclear debris from fragmented neutrophils. Fibrinoid necrosis was not identified.

MPO-ANCA was elevated at 16.4 IU/mL (reference range <3.5 IU/L). No elevations in SP-A or KL-6 were noted, and tests for anti-ARS, anti-MDA5, anti-Mi-2, and anti-TIF1-γ antibodies were all negative. Blood cultures were negative, and no findings suggestive of infection were observed. Nerve conduction studies revealed an axonal degeneration pattern in the bilateral lower limbs. A skin biopsy of the palpable purpura on the medial left lower leg, which developed five days after the initial visit, was performed on day 11. The biopsy demonstrated leukocytoclastic vasculitis within the dermis, indicating vasculitis (Figure 3).

According to the 2022 American College of Rheumatology/European Alliance of Associations for Rheumatology (ACR/EULAR) classification criteria, the patient scored 9 points (MPO-ANCA positivity and interstitial lung disease, without PR3-ANCA, sinonasal disease, or eosinophilia), supporting the diagnosis of MPA [10]. Based on these findings, microscopic polyangiitis (MPA) was diagnosed. Treatment with prednisolone was initiated on day 13, at a dose of 30 mg and subsequently tapered; 10 months later, the patient remains on 6 mg daily. Azathioprine 50 mg has also been continued for 10 months. Erythrocyte sedimentation rate (ESR) was measured only at the initial visit and was elevated. For subsequent evaluation of treatment response and follow-up, we primarily monitored C-reactive protein (CRP) levels, supplemented by MPO-ANCA as an adjunctive marker (Table 2).

**Table 2:** For subsequent evaluation of treatment response and follow-up, we primarily monitored C-reactive protein (CRP) levels, supplemented by MPO-ANCA as an adjunctive marker Treatment was initiated on day 13.

	At presentation, Day 1	Day 6	Day 13	Day 20	Day 41 (6 weeks later)	Day 160 (5 months later)	Reference range (adults)
C-reactive protein (mg/dl)	8.81	8.38	9.06	2.00	0.78	0.04	< 0.14
Myeloperoxidase anti-neutrophil cytoplasmic antibody (MPO-ANCA) (IU/ml)	16.4				5.0	3.0	< 3.5

Both CRP and MPO-ANCA progressively declined over time, consistent with suppression of disease activity. The skin lesions resolved one week after the initiation of treatment. By four weeks, cough and dyspnea had disappeared, and the numbness showed signs of improvement. Pregabalin and mirogabalin were co-administered for the neuropathy, and the affected area gradually diminished over time, leaving only a small residual area on the left sole after six months.

## Discussion

MPA is the most common subtype of ANCA-associated vasculitis (AAV), accounting for approximately 50% of cases [3]. The incidence of MPA is notably higher in Japan compared to Western countries [11]. MPA is a pauci-immune, necrotizing, small-vessel vasculitis strongly associated with MPO-ANCA. MPO-ANCA activates neutrophils, leading to endothelial injury and leukocytoclastic vasculitis [1,2]. MPA is characterized by systemic manifestations, such as fever and weight loss, along with localized organ involvement, including rapidly progressive glomerulonephritis in the kidneys and alveolar hemorrhage or interstitial pneumonia in the lungs [12]. Additionally, it may present with purpura, mononeuritis multiplex, arthralgia, and myalgia [12]. Approximately 30% of patients with MPA develop dermatologic manifestations, including purpura, skin ulcers, livedo reticularis, and subcutaneous nodules, with purpura being the most frequently observed [13]. Histopathologically, purpura and leukocytoclastic vasculitis are common to all AAVs; however, inflammation in MPA tends to extend into deeper layers of the skin compared with other AAVs, and extravascular granulomas are generally absent [2]. Cutaneous small vessel vasculitis in MPA typically involves dependent areas, most often the lower extremities, and, in bedridden patients, the buttocks [14].

Cutaneous manifestations can be the initial feature of MPA in a notable subset of patients, reported in approximately 15-30% of cases [15-16]. While MPA often follows a rapidly progressive course, atypical presentations have also been described, including cases with a slowly progressive clinical course [17]. Furthermore, a cutaneous-limited form of MPO-ANCA-positive MPA has been reported, characterized by recurrent crops of purpura over weeks to months [18]. These observations highlight the heterogeneity of MPA and underscore the diagnostic importance of cutaneous findings. In addition, although initially unilateral purpura is extremely rare, a recent report described a case of cutaneous-limited, strongly unilateral MPA, in which purpura appeared on one side at diagnosis and only developed on the contralateral side approximately one year later [19]. This further emphasizes the variable and sometimes localized cutaneous presentations of MPA and the need for careful longitudinal observation.

In the differential diagnosis of the reticular and fibrotic changes observed on chest CT, idiopathic pulmonary fibrosis (IPF), connective tissue disease-associated interstitial lung disease (CTD-ILD), and asbestosis were considered. The patient had muscle weakness but no cutaneous manifestations or elevated creatine kinase; nevertheless, anti-aminoacyl tRNA synthetase (anti-ARS) syndrome and MDA5-positive dermatomyositis were also evaluated. However, all relevant autoantibodies were negative, and no evidence of drug-induced pneumonitis, diabetic lung, or infectious pneumonia was found. Based on these findings, the subpleural basal honeycombing was interpreted as MPA-associated interstitial lung disease. Although such honeycombing is generally indicative of a chronic fibrotic process rather than acute vasculitic changes, this interpretation aligns with previous reports showing that MPO-ANCA-positive patients frequently develop interstitial pneumonia and pulmonary fibrosis, whereas PR3-ANCA-positive patients more commonly present with pulmonary nodules, cavitary lesions, and ear-nose-throat involvement. Importantly, several studies have demonstrated that usual interstitial pneumonia (UIP) is the most prevalent histopathological pattern in MPA-associated interstitial pneumonia, followed by nonspecific interstitial pneumonia (NSIP) [20-23]. Our patient also exhibited a UIP pattern, consistent with these findings.

In this case, initial dermatologic findings were confined to localized purpura on the right lower extremity. However, the subsequent emergence of palpable purpura contributed to the diagnosis of vasculitis. Differential diagnoses of palpable purpura include drug-induced vasculitis, infection-related vasculitis, immune complex vasculitis (such as IgA vasculitis), and connective tissue disease-related vasculitis. This approach is consistent with practical reviews noting that cutaneous small-vessel vasculitis most often presents as symmetrically distributed palpable purpura of the lower extremities and may arise secondary to infection, medication, connective tissue disease, or malignancy [24]. In our patient, the absence of systemic infection, drug exposure history, and immune complex deposition on biopsy supported the diagnosis of MPA. This case illustrates the diagnostic value of cutaneous manifestations in ANCA-associated vasculitis. Recognition of progressive purpura enabled the timely biopsy and early diagnosis of MPA, even before significant renal or pulmonary involvement became evident.

## Conclusions

In this case, a diagnosis of microscopic polyangiitis (MPA) was established based on the clinical course involving symptoms such as dry cough and myalgia, followed by the emergence of painful purpura and peripheral neuropathy. A combination of findings, including MPO-ANCA positivity, skin biopsy, and nerve conduction studies, enabled early diagnosis and prompt initiation of treatment. The most significant feature of this case was that cutaneous findings played a decisive role in the diagnosis. Purpura is easily visible and often appears early in the disease course, serving as a useful clue when evaluating patients with non-specific systemic symptoms. In conclusion, rapidly progressive cutaneous findings, even in the absence of prominent renal or pulmonary manifestations, should alert clinicians to the possibility of MPA. Careful assessment of skin lesions can therefore play a decisive role in facilitating an early diagnosis and appropriate management of this disease. Clinicians should remain alert to atypical and localized skin findings, as their careful assessment may allow timely diagnosis of MPA and prevent progression to life-threatening organ involvement.
